# Supplementary material for: HATCHet2: clone- and haplotype-specific copy number inference from bulk tumor sequencing data
Source: Genome Biol. 2024 May 21;25:130. doi: 10.1186/s13059-024-03267-x (PMC11110434; doi:10.1186/s13059-024-03267-x)
Supplement: Supplementary file 1 — Additional file 1. Supplement. Supplemental methods sections S1-S10 and supplemental figures S1-S14. [file 13059_2024_3267_MOESM1_ESM.pdf]

Supplemental Information for "HATCHet2: clone- and haplotype-specific copy number inference from bulk tumor sequencing data"

Contents

S1 Benchmarking HATCHet2 against HATCHet [1], Battenberg [2], cloneHD [3], and TITAN [4] 2

S1.1 Overview . . . 2

S1.2 Metrics for evaluating performance on simulated data . . . 2

S2 Calculation of mean squared error for HATCHet2 and Battenberg 2

S3 Reference-based phasing 3

S3.1 Implementation details . . . 3

S3.2 Construction of phasing blocks . . . 3

S4 Haplotype switch correction algorithm 3

S5 Additional evaluation on MASCoTE simulated data 4

S5.1 Performance on simulated data as a function of segment size . . . 4

S5.2 Performance in inferring simulated tumor clones and clone proportions . . . 4

S6 Generation of simulated data with varying purity 4

S7 Evaluating HATCHet2 and HATCHet on simulated data with varying purity. 5

S8 Generation of simulated data with mirrored-subclonal CNAs. 5

S9 Evaluating HATCHet2 and HATCHet on simulated data with mirrored-subclonal CNAs. 6

S10 Focal amplifications identified by HATCHet2, HATCHet [1], and Battenberg [2] on prostate cancer patients. 6

S1 Benchmarking HATCHet2 against HATCHet [1], Battenberg [2], cloneHD [3], and TITAN [4]

S1.1 Overview

Simulated data was generated using the MASCoTE simulator [1] which simulates sequencing reads from *in silico* mixtures of synthetic tumor genomes. We used the 8 multi-sample datasets included in the HATCHet publication [1], which include four datasets with 3 bulk samples each and four with 5 bulk samples each. Each sample is a different mixture of the 2-3 tumor genomes (i.e., clones) simulated for that individual. Half of the datasets include a whole-genome duplication affecting all tumor clones.

S1.2 Metrics for evaluating performance on simulated data

We used several different metrics to quantify different aspects of performance in recovering CNAs from simulated data. Let $\mathcal{S}_s$ indicate the set of haplotype-specific copy-number states that a method infers for a particular genomic segment s across the n tumor clones:

$$\mathcal{S}_s = \{(a_{s,i}, b_{s,i}) | 1 \leq i \leq n\} \tag{S0.1}$$

Let $\mathcal{T}_s$ indicate the set of true haplotype-specific copy-number states for region s. Let ℓ_s indicate the length of genomic segment s, and let $L = \sum_s \ell_s$ be the total length of all genomic segments. Then, the precision, recall, and accuracy of haplotype-specific copy-number states is as follows:

$$\text{precision}(\mathcal{S}_s) = \frac{1}{L}\sum_s \ell_s \frac{|\mathcal{S}_s \cap \mathcal{T}_s|}{|\mathcal{S}_s|} \tag{S0.2}$$

$$\text{recall}(\mathcal{S}_s) = \frac{1}{L}\sum_s \ell_s \frac{|\mathcal{S}_s \cap \mathcal{T}_s|}{|\mathcal{T}_s|} \tag{S0.3}$$

$$\text{accuracy}(\mathcal{S}_s) = \frac{1}{L}\sum_s \ell_s \frac{|\mathcal{S}_s \cap \mathcal{T}_s|}{|\mathcal{S}_s \cup \mathcal{T}_s|} \tag{S0.4}$$

Following [1], we compute the average allele-specific error per genome position (AASEGP) by considering the clone mixture proportions as a probability distribution over discrete copy-number states and computing the total variation distance between the true and inferred distributions. Specifically, let $u_{s,c,p}$ indicate the proportion of cells in sample p that are assigned haplotype-specific copy-number state c in segment s. Let $\overline{u}_{s,c,p}$ indicate the true value of the corresponding quantity. Let P indicate the number of samples. Then,

$$AASEGP(U) = \frac{1}{LP}\sum_s \sum_{p=1}^{P} \ell_s \max_{c \in \mathcal{S}_s \cup \mathcal{T}_s} |u_{s,c,p} - \overline{u}_{s,c,p}| \tag{S0.5}$$

We report the average allele-specific *accuracy* per genome position $AASAPGP(U) = 1 - AASEPGP(U)$ so that “higher is better" for all metrics used for comparison on the simulated data.

The results reported for Battenberg [2], cloneHD [3], and TITAN [4] on these simulated datasets are the same as those reported in the original HATCHet publication [1].

S2 Calculation of mean squared error for HATCHet2 and Battenberg

For each method, we computed the mean squared error (MSE) for both BAF and RDR by comparing the expected RDR and BAF of each method's solution (a function of the assigned copy-number states as well as the inferred sample purity and tumor genome length) to the observed BAF and RDR values within the region, taking the difference, and squaring it. Specifically, we evaluated the BAF error at every SNP position

in the region and the RDR error at every interval between SNP positions – i.e., given SNPs at positions i and j, we computed the RDR in the genomic interval $[i, j)$ in each sample and evaluated the MSE comparing this RDR to the expected RDR.

S3 Reference-based phasing

S3.1 Implementation details

By default, HATCHet2 downloads a 1000 Genomes Project [5] (phase 3, version 5) reference panel that contains haplotypes in hg19 coordinates. To support the phasing of germline SNPs in hg38 coordinates, HATCHet2 also downloads chain files from the UCSC genome browser (http://genome.ucsc.edu) and uses Picard [6] (https://broadinstitute.github.io/picard/) to liftover between the hg19 and hg38 human reference genome versions, both before and after reference-based phasing. HATCHet2 then filters out multi-allelic SNPs and indels using bcftools [7] and phases the resulting bi-allelic germline SNPs with SHAPEIT2 [8].

S3.2 Construction of phasing blocks

Given reference-based phasing output, we first iterate over SNPs and apply a test between pairs of adjacent SNPs. Let x_i indicate the number of reads assigned to haplotype "1" at SNP i and y_i indicate the number of reads assigned to haplotype "0" at SNP i (where assignment to haplotypes is given by the reference-based phasing). For each pair i, j of adjacent SNPs, we test whether the binomial proportion $x_i/(x_i+y_i)$ is different from the proportion $x_j/(x_j + y_j)$ at significance level α (default 0.1) using the normal approximation test. Then, we group SNPs to minimize the total number of phasing blocks such that

1. Phasing blocks are contiguous and non-overlapping along the genome.
2. Adjacent SNPs i, j that were significantly different according to the binomial proportion test are not combined.
3. Adjacent SNPs i, j that are more than a distance of d (default 25 kb) apart are not combined in a phasing block.
4. No more than c SNPs (default 10) are combined in a block.

We solve this problem greedily by iterating over SNPs in each bin and forming maximal phasing blocks subject to the above constraints. We merge the counts for all SNPs in each resulting phasing block to form a "meta-SNP" as described in the main text.

S4 Haplotype switch correction algorithm

While we infer the relative phase for SNPs in each bin when estimating the mhBAF, the relative phase *across* bins may not be consistent, particularly in bins where both haplotypes are approximately in equal abundance (i.e., the "minor" haplotype for the corresponding section of the genome is somewhat ambiguous). In these regions, the minor haplotype may "switch" between adjacent bins, which would result in mhBAF values being reflected about 0.5 in each sample. To ensure that the minor haplotype is consistent across bins on each chromosome arm, we apply the following algorithm to identify and correct haplotype switches. Let $\hat{f}_{s,p}$ indicate the inferred mhBAF value for bin $s \in [1, N]$ and sample $p \in [1, P]$ on a chromosome arm with N bins.

1. Identify samples p for which the average allelic imbalance $\frac{1}{N}\sum_{s=1}^{N} |\hat{f}_{s,p} - 0.5|$ is large (at least 0.02 by default). Let $\mathcal{A}$ refer to the set of such samples. Observe that samples without substantial allelic imbalance are not informative for identifying haplotype switches.
2. Identify haplotype switches as the set $\mathcal{L}$ of bins s where $\text{sign}(\hat{f}_{s-1,p} - 0.5) \neq \text{sign}(\hat{f}_{s,p} - 0.5)$ for *all* samples $s \in \mathcal{A}$.
3. Compute the proportion $|\mathcal{L}|/(N-1)$ of haplotype switches on the chromosome arm. If this proportion does not exceed a threshold (default 0.01), then do not correct haplotype switches.

4. If sufficiently many haplotype switches are detected, perform piecewise constant regression on the average haplotype frequencies across imbalanced samples. By default, we fit 10 segments to each chromosome arm.
5. Identify those segments that have a) sufficiently many haplotype switches (default $\geq 10\%$); b) sufficiently high average mhBAF (default ≥ 0.45); and c) sufficiently high allelic imbalance in at least 1 sample (default ≥ 0.02). For each such segment, assign haplotypes to each bin to minimize the pairwise difference in mhBAF between adjacent bins in the sample with the most extreme allelic imbalance (on average for the segment).

S5 Additional evaluation on MASCoTE simulated data

S5.1 Performance on simulated data as a function of segment size

To demonstrate the performance of HATCHet2 in inferring copy-number events of varying sizes, we evaluated the recall and precision of HATCHet2, HATCHet [1], cloneHD [3], TITAN [4], and Battenberg [2] on the MASCoTE simulated multi-sample datasets described in Results. HATCHet2 and HATCHet outperformed the best of the other 3 methods across size ranges on both methods (average improvement in precision by 133% and recall by 78% vs. the third-best method for each size range and dataset), while the HATCHet2 and HATCHet performed comparably (improvement in precision by 1.7% and decrease in recall by 0.006%; Fig. S9A-B). We then compared HATCHet2 to HATCHet on single-sample datasets by considering each MASCoTE simulated sample independently, and found that HATCHet2 outperformed HATCHet by a larger margin (6.2% higher precision, 5.7% higher recall; Fig. S9C-D). Moreover, on segments smaller than 1 Mb, HATCHet2 achieved 16.6% higher precision and 15.2% higher recall than HATCHet.

S5.2 Performance in inferring simulated tumor clones and clone proportions

To evaluate how well HATCHet2 recovers the number and proportion of tumor clones, we evaluated the number and proportions of the clones inferred by HATCHet2, HATCHet [1], cloneHD [3], TITAN [4], and Battenberg [2] on the MASCoTE simulated datasets described in Results. Each method inferred the correct number of tumor clones in about half of instances, except TITAN which systematically underestimated the number of tumor clones (Fig. S10A). We then evaluated the performance of each method in inferring the clone proportions on the subset of datasets where the method correctly inferred the number of clones (as such, TITAN was excluded from this analysis). For all methods, we evaluated each inferred tumor clone against the true tumor clone which most closely matched its proportions. HATCHet2 and HATCHet outperformed the other methods in terms of both mean absolute difference in clone proportions and total variation distance (by $13\times$ and $9.5\times$, respectively; Fig. S10B-C). HATCHet2 performed similarly to HATCHet on these instances, with a 2.4% higher mean absolute error and a 5% lower total variation distance.

We further evaluated the performance of HATCHet2 against HATCHet treating each sample as an independent dataset ($n = 32$). In this setting, both methods were fixed to the correct number of tumor clones, so we evaluated all instances. In this setting, HATCHet2 outperformed HATCHet in both metrics, with a 29.9% lower mean absolute difference and 27.3% lower total variation distance (Fig. S10D-E).

S6 Generation of simulated data with varying purity

The procedure we used to generate simulated datasets with varying purity was as follows:

1. Select a 2-3 clone tumor genome generated by MASCoTE [1] from the HATCHet publication repository [9].
2. Remove 25% of segments uniformly at random, then insert 10 events each of the following sizes: 10 Kb, 50 Kb, 200 Kb, 500 Kb. The states assigned to inserted events are sampled uniformly at random from existing clone-specific copy-number states present in the original genome.

3. Add mirrored events: for 50% of inserted events and 25% the remaining segments, swap the two haplotypes for a single clone. Note that due to the abundance of balanced segments, the resulting tumor genome will have considerably less than 25% mirrored segments.
4. For each simulated sample tumor s, sample the coverage c_s Poisson(80). For the matched normal sample, sample the coverage from Poisson(30).
5. Sample the total reads for each region from a Poisson distribution with expected value proportional to the fractional copy number of the region.
6. For each SNP, sample total reads from a Poisson distribution proportional to the overlapping region's fractional copy number, then sample alternate reads from a binomial distribution parameterized by the true BAF for the overlapping region.

Given interval total reads and SNP-covering reads, we then ran HATCHet2 starting with the binning step. The code used to generate simulated data is available in the simulation repository [10]. The results and detailed parameter settings used in these simulations are available in the publication repository [11].

S7 Evaluating HATCHet2 and HATCHet on simulated data with varying purity.

To evaluate the capability of HATCHet2 to detect tumor clones in low-purity samples, we simulated 191 2-sample datasets intended to emulate a liquid biopsy setting in which one "solid" tumor sample had high purity (0.9) and the second "liquid" sample had variable low purity.In the liquid sample, all 2-3 tumor clones were present at the same proportion ranging from 0.05 to 0.3. This design was meant to emulate a research setting in which an initial high-purity sample was sequenced along with one (or potentially more) liquid biopsies at lower purity.

We found that while HATCHet2 performed poorly on the datasets in which the liquid sample clone proportion was 0.05, it was able to recover the tumor clones at proportions 0.1 and higher (Fig. S14). At the upper end of the tested clone proportion range, performance began to deteriorate as HATCHet2 struggled to differentiate between the multiple tumor clones present at the same proportion.

S8 Generation of simulated data with mirrored-subclonal CNAs.

We generated simulated data with mirrored-subclonal CNAs similarly to the approach described in section S6 , but using different simulated genomes and clone proportions. Specifically, we generated genomes using the following steps:

1. Create a template genome by starting with the MASCoTE simulated genome "dataset_n2_s14855" and removing 80% of segment endpoints, to create a list of 117 segments across the genome.
2. Sample copy-number states at random to populate the segments in the genome. For each segment, we first decided whether the state would be clonal, subclonal, or mirrored, with probabilities $0.5, 0.2$, and 0.3, respectively. Subclonal and mirrored-subclonal states were sampled uniformly at random from a list of candidate states. Clonal states were sampled at random from a list of candidate states, with extra probability mass was placed on the "characteristic" clonal state(s) that HATCHet2 and HATCHet use to determine purity: $(1, 1)$ for diploid genomes, and $(2, 2)$ and $(2, 1)$ for tetraploid genomes. The remaining candidate clonal states were weighted uniformly.

Each of the mirrored-subclonal simulated datasets consisted of two clones. The clone proportions we used to generate the mirrored-subclonal datasets were as follows, where the first number in each sample describes the proportion of normal cells, then the proportion for clone 1, then the proportion for clone 2.

1. 3 samples: $(0.1, 0.6, 0.3), (0.3, 0, 0.7), (0.1, 0.9, 0)$
2. 2 samples: $(0.2, 8, 0), (0.5, 0, 0.5)$
3. 2 samples: $(0.1, 0.9, 0), (0.1, 0.35, 0, 0.65)$

Note that each dataset includes two samples that each contain only one clone.

S9 Evaluating HATCHet2 and HATCHet on simulated data with mirrored-subclonal CNAs.

To evaluate the performance of HATCHet2 in recovering mirrored-subclonal CNAs, we generated a total of 150 simulated datasets as described in Section S8. These simulated datasets include all combinations of 10 simulated genomes (5 diploid, 5 tetraploid) with the 3 sets of clone proportions described above, using 5 different random number generator seeds for each combination. We applied HATCHet2 and HATCHet to these simulated datasets and measured the AASAPGP and accuracy, both on the whole genomes and restricted to mirrored-subclonal events (i.e., measuring performance on only those states (A, B) where $B > A$) (Fig. S11). We refer to these restricted metrics as "mirrored accuracy" and "mirrored AASAPGP", respectively. HATCHet2 outperformed HATCHet in all of these metrics ($p < 10^{-14}$, average improvement $\geq 30.6\%$) and especially in terms of mirrored-subclonal states ($p < 10^{-62}$, average improvement $\geq 1100\%$). HATCHet performs especially poorly on 3 of the tetraploid genomes, with an average AASAPGP of 0.36 and average accuracy of 0.13, compared to 0.92 and 0.86, respectively, for HATCHet2. Indeed, HATCHet failed to identify any mirrored-subclonal CNAs in 140/150 instances, with the exceptions being diploid genomes 1 and 2 with clone proportions 1.

To further analyze the difference in performance on mirrored-subclonal CNAs, we examined the HATCHet2 and HATCHet results from two simulated datasets in detail: one dataset in which HATCHet was able to identify some mirrored-subclonal CNAs (with diploid genome 1, sample clone proportions 1, and random seed 0; Fig. S11E-J), and one in which it was not (with the same diploid genome 1, sample clone proportions 2, and random seed 0; Fig. S11K-N). In the first dataset, the two clusters containing mirrored-subclonal CNAs (denoted by red circles) have mhBAF below 0.5 in the first sample (Fig. S11E,H), so the mBAF used by HATCHet is equal to the mhBAF used by HATCHet2 for these clusters in this sample and thus HATCHet is able to identify the mirrored-subclonal states (mirrored accuracy 0.992). However, since HATCHet does not retain haplotype information across samples, HATCHet is unable to assign the mirrored-subclonal states correctly to clones and thus incorrectly infers that both of the other samples contain the same clone (mirrored AASAPGP 0.3; Fig. S11I-J). In contrast, HATCHet2 using the mhBAF correctly assigns the mirrored-subclonal states to distinct clones in the single-clone samples (mirrored accuracy 0.997, mirrored AASAPGP 0.988; Fig. S11F-G). The second dataset consists of the same simulated genome (i.e., the same copy-number states in all segments for both clones) as the first example dataset, but with different sample clone proportions. In this case, HATCHet2 recovers the mirrored-subclonal CNAs in both clones (mirrored accuracy 0.996, mirrored AASAPGP 0.915; Fig. S11K-L), but HATCHet is unable to distinguish between events affecting different haplotypes and thus identifies no mirrored-subclonal CNAs (mirrored accuracy 0, mirrored AASAPGP 0; Fig. S11M-N).

S10 Focal amplifications identified by HATCHet2, HATCHet [1], and Battenberg [2] on prostate cancer patients.

We analyzed the focal amplifications identified by HATCHet2, HATCHet [1], and Battenberg [2] in multi-sample WGS data from 10 prostate cancer patients from Gundem et al., 2015 [12]. Particularly, for each method, we counted the number of segments smaller than 1 Mb for which the corresponding method assigned a total copy number of at least 4 to at least 1 tumor clone. In keeping with our finding in the main text that HATCHet infers more and shorter segments than HATCHet2 and Battenberg infers fewer and longer segments than HATCHet2, we find that HATCHet2 identifies fewer focal amplifications than HATCHet but more than Battenberg (Fig. S12A).

Next, we focused on the focal amplifications called by HATCHet2 and counted the proportion of these amplifications that overlapped focal amplifications called by other methods. We found that on average, 48.7% of the focal amplifications called by HATCHet2 were also called by either HATCHet, Battenberg, or both

(Fig. S12B). Interestingly, this varied highly from patient to patient, with as many as 86% of HATCHet2's focal amplifications shared for patient A21 and as few as 3.4% shared for patient A29. HATCHet2 called more high amplifications for patient A29 than for nearly all other patients (Fig. S13), which other methods may have missed.

Finally, we assessed the overlap between the inferred focal amplifications and the 41 TCGA prostate cancer genes from [13] included in Fig. 2B. While HATCHet and HATCHet2 often infer focally amplified TCGA genes in these highly aneuploid tumors (7.8 and 7.9 genes on average, respectively), Battenberg rarely identifies any (0.8 genes on average; Fig. S12C).

Author details

References

1. Zaccaria, S., Raphael, B.J.: Accurate quantification of copy-number aberrations and whole-genome duplications in multi-sample tumor sequencing data. Nature communications **11**(1), 1–13 (2020)
2. Nik-Zainal, S., Van Loo, P., Wedge, D.C., Alexandrov, L.B., Greenman, C.D., Lau, K.W., Raine, K., Jones, D., Marshall, J., Ramakrishna, M., *et al.*: The life history of 21 breast cancers. Cell **149**(5), 994–1007 (2012)
3. Fischer, A., Vázquez-García, I., Illingworth, C.J., Mustonen, V.: High-definition reconstruction of clonal composition in cancer. Cell reports **7**(5), 1740–1752 (2014)
4. Ha, G., Roth, A., Khattra, J., Ho, J., Yap, D., Prentice, L.M., Melnyk, N., McPherson, A., Bashashati, A., Laks, E., *et al.*: Titan: inference of copy number architectures in clonal cell populations from tumor whole-genome sequence data. Genome research **24**(11), 1881–1893 (2014)
5. Consortium, .G.P., *et al.*: A global reference for human genetic variation. Nature **526**(7571), 68 (2015)
6. Picard toolkit. Broad Institute (2019)
7. Danecek, P., Bonfield, J.K., Liddle, J., Marshall, J., Ohan, V., Pollard, M.O., Whitwham, A., Keane, T., McCarthy, S.A., Davies, R.M., *et al.*: Twelve years of samtools and bcftools. Gigascience **10**(2), 008 (2021)
8. Delaneau, O., Zagury, J.-F., Robinson, M.R., Marchini, J.L., Dermitzakis, E.T.: Accurate, scalable and integrative haplotype estimation. Nature communications **10**(1), 5436 (2019)
9. Zaccaria, S.: HATCHet publication repository. GitHub (2020). https://github.com/raphael-group/hatchet-paper
10. Myers, M.: HATCHet2 publication repository. GitHub (2024). https://github.com/raphael-group/hatchet2-simulations
11. Myers, M.: HATCHet2 publication repository. GitHub (2024). https://github.com/raphael-group/hatchet2-paper
12. Gundem, G., Van Loo, P., Kremeyer, B., Alexandrov, L.B., Tubio, J., Papaemmanuil, E., Brewer, D.S., Kallio, H.M., Högnäs, G., Annala, M., *et al.*: The evolutionary history of lethal metastatic prostate cancer. Nature **520**(7547), 353–357 (2015)
13. Abeshouse, A., Ahn, J., Akbani, R., Ally, A., Amin, S., Andry, C.D., Annala, M., Aprikian, A., Armenia, J., Arora, A., *et al.*: The molecular taxonomy of primary prostate cancer. Cell **163**(4), 1011–1025 (2015)
14. Sondka, Z., Bamford, S., Cole, C.G., Ward, S.A., Dunham, I., Forbes, S.A.: The cosmic cancer gene census: describing genetic dysfunction across all human cancers. Nature Reviews Cancer **18**(11), 696–705 (2018)
15. Zaccaria, S., Raphael, B.J.: Characterizing allele-and haplotype-specific copy numbers in single cells with chisel. Nature biotechnology **39**(2), 207–214 (2021)

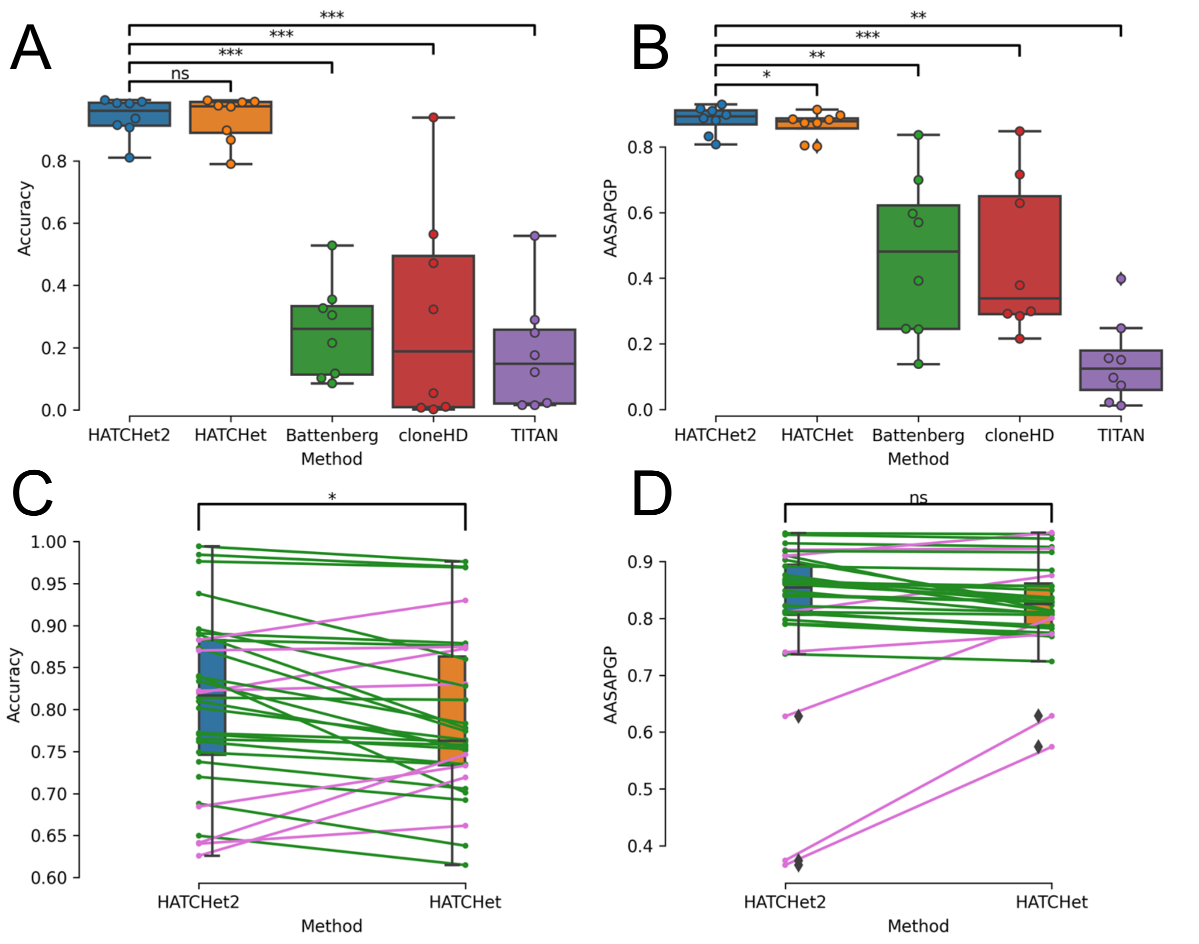

Fig. S1: Benchmarking HATCHet2, HATCHet [1], TITAN [4], cloneHD [3], and Battenberg [2] on simulated data. **A-B** Results from all methods on 8 multi-sample datasets simulated using MASCoTE [1] measured by accuracy (A) and AASAPGP (B). For all panels, asterisks indicate significance by paired t-test: $* = 0.01 \leq p < 0.05$, $** = 10^{-3} \leq p < 0.01$, $*** = p < 10^{-3}$. **C-D** Results from HATCHet2 and HATCHet [1] on the same data with each sample run independently ($n = 32$ samples), again measured by accuracy (C) and AASAPGP (D). Green lines indicate datasets on which HATCHet2 achieved a higher accuracy than HATCHet, whereas pink lines indicate that HATCHet achieved a higher accuracy.

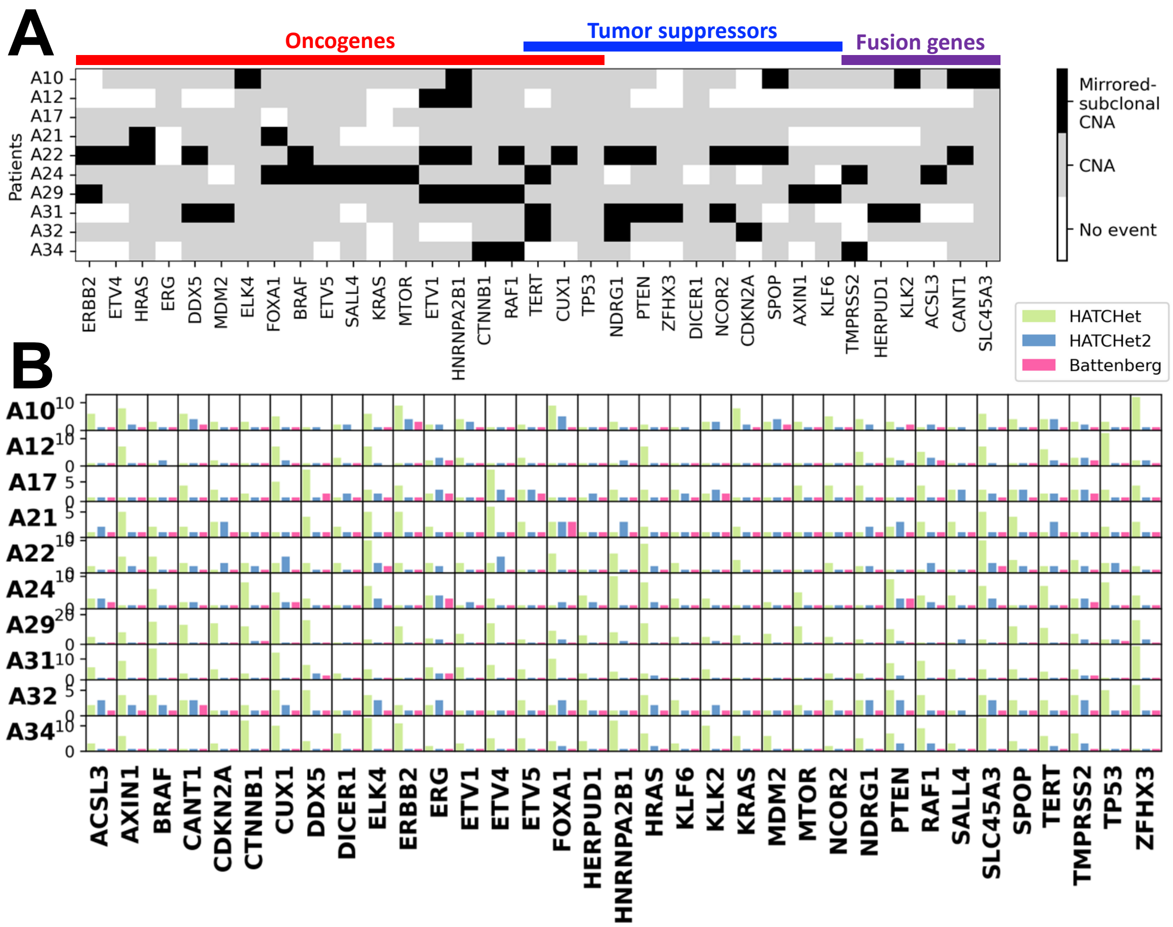

Fig. S2: Focal copy-number changes called by HATCHet2, HATCHet [1], and Battenberg [2] near prostate cancer genes. **A** Number of segments identified by each method for each patient (row) within 1 megabase of 35 genes (columns) from the Cancer Gene Census (CGC) [14]. **B** Copy-number landscape for 35 prostate-cancer-related genes from COSMIC Cancer Gene Census [14] across 10 prostate cancer patients from [12]. White entries indicate that no copy-number aberration was identified at the locus. Black entries indicate that mirrored-subclonal CNAs were observed among the tumor clones at the locus. Gray entries indicate that a non-mirrored copy-number aberration was inferred for at least one clone at the locus.

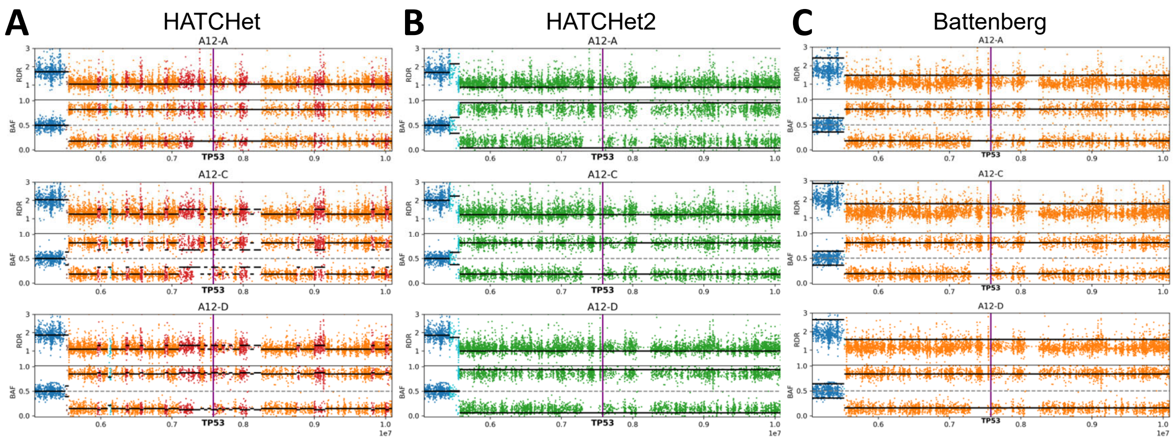

Fig. S3: Segments from HATCHet2, HATCHet [1], and Battenberg [2] near TP53 locus on chromosome 17 in patient A12. As in Fig. 2, each point is a small region between adjacent SNPs (RDR) or a SNP (BAF) colored by the assigned copy-number state of the overlapping segment in the corresponding solution. Black bars indicate the expected RDR and BAF of each segment according to the copy-number states and clone proportions identified by the corresponding method. Genes are represented as purple bars. **A** Solution from HATCHet. **B** Solution from HATCHet2. **C** Solution from Battenberg.

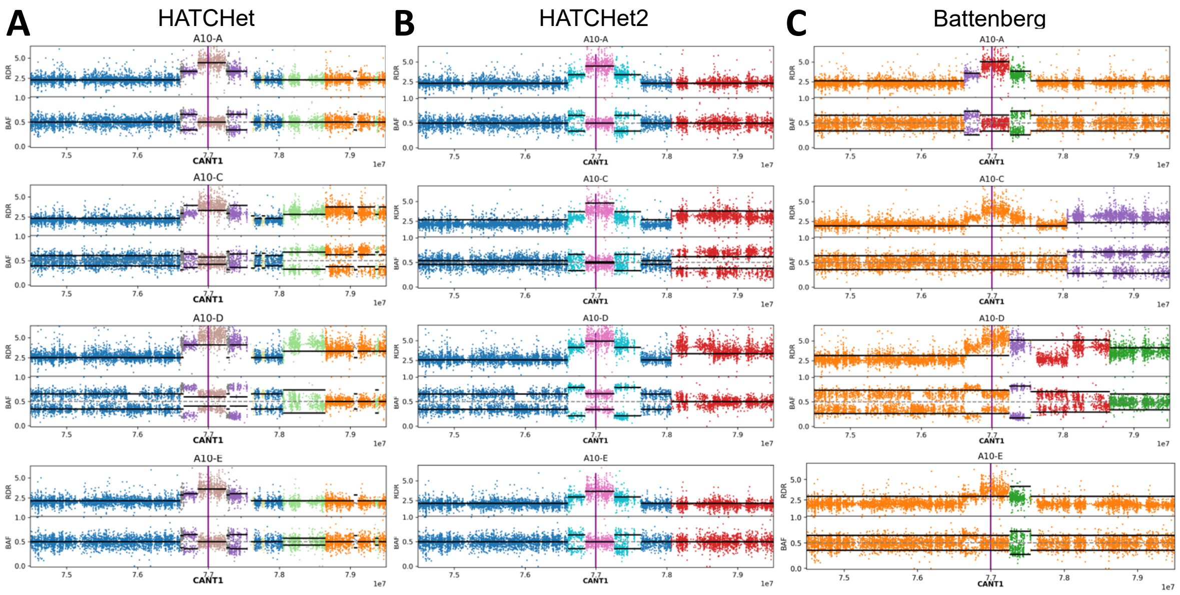

Fig. S4: Segments from HATCHet2, HATCHet [1], and Battenberg [2] near CANT1 locus on chromosome 17 in patient A10. As in Fig. 2, each point is a small region between adjacent SNPs (RDR) or a SNP (BAF) colored by the assigned copy-number state of the overlapping segment in the corresponding solution. Black bars indicate the expected RDR and BAF of each segment according to the copy-number states and clone proportions identified by the corresponding method. Genes are represented as purple bars. **A** Solution from HATCHet. **B** Solution from HATCHet2. **C** Solution from Battenberg.

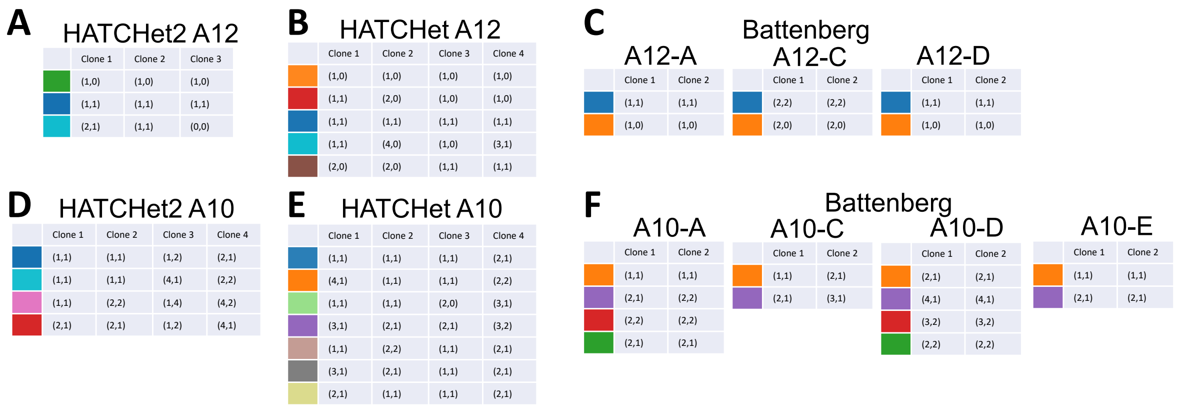

Fig. S5: Copy-number state legends for HATCHet2, HATCHet [1], and Battenberg [2] focal copy number panels. For HATCHet2 and HATCHet, all colors for the same patient represent the same copy-number state. For Battenberg, which calls each sample independently, the colors do not correspond between samples for the same patient. **A** Copy-number state legend for HATCHet2 patient A12 figure panels showing the region near TP53 (Fig. 2C and Fig. S3). **B** Copy-number state legend for HATCHet patient A12 figure panels showing the region near TP53 (Fig. 2D and Fig. S3). **C** Copy-number state legend for Battenberg patient A12 figure panels showing the region near TP53 (Fig. S3). **D** Copy-number state legend for HATCHet2 patient A10 figure panels showing the region near CAN1 (Fig. 2E and Fig. S4). **E** Copy-number state legend for HATCHet patient A10 figure panels showing the region near CAN1 (Fig. S4). **F** Copy-number state legend for HATCHet2 patient A10 figure panels showing the region near CAN1 (Fig. 2F and Fig. S4).

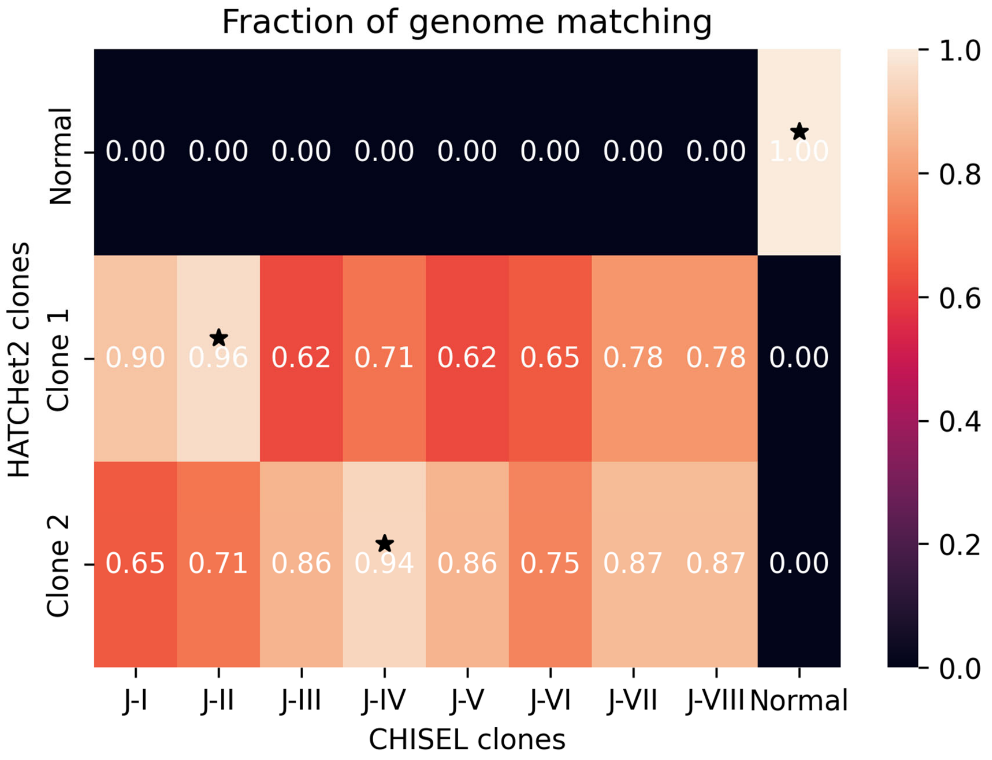

Fig. S6: Comparison between HATCHet2 clones and CHISEL [15] clones from 10X single-cell whole-genome sequencing data. Proportion of the genome with the same haplotype-specific copy-number assignment between HATCHet2 clones (rows) inferred from pseudo-bulk samples and CHISEL clones (columns) inferred by aggregating single cells. Stars indicate the CHISEL clone which most closely matches each HATCHet2 clone.

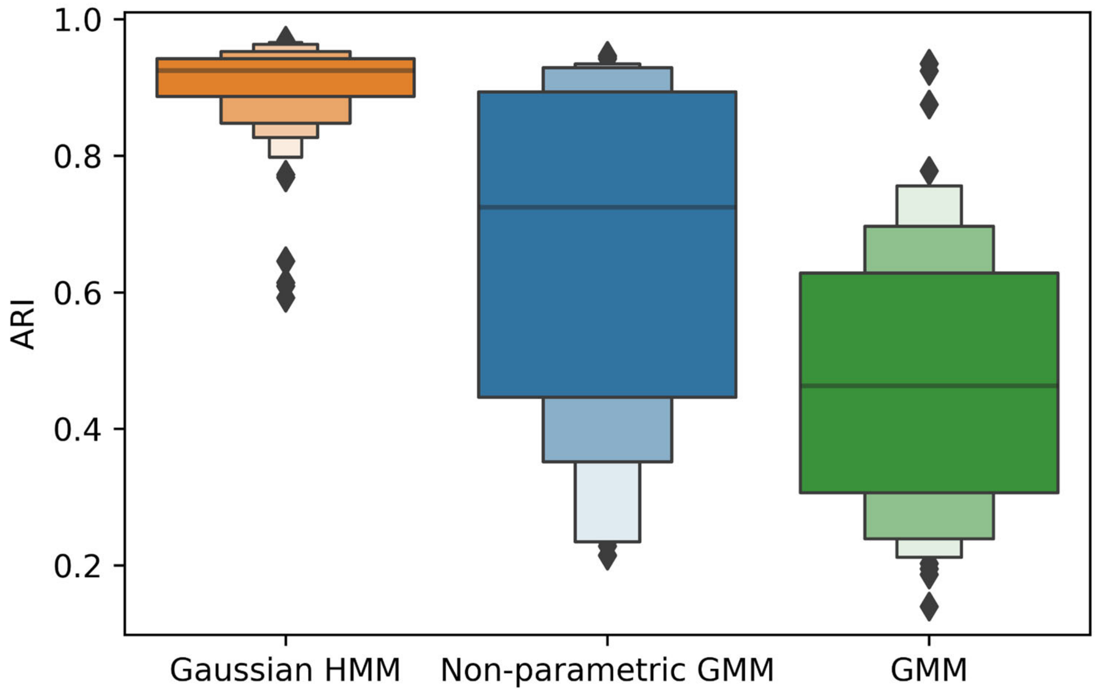

Fig. S7: Comparison of clustering methods on simulated data. Comparison of clustering performance for 3 different methods in recovering distinct copy-number states from 64 simulated generated using MASCoTE [1]. Y-axis shows the adjusted Rand index (ARI) when comparing the inferred clusters to the ground truth copy-number events.

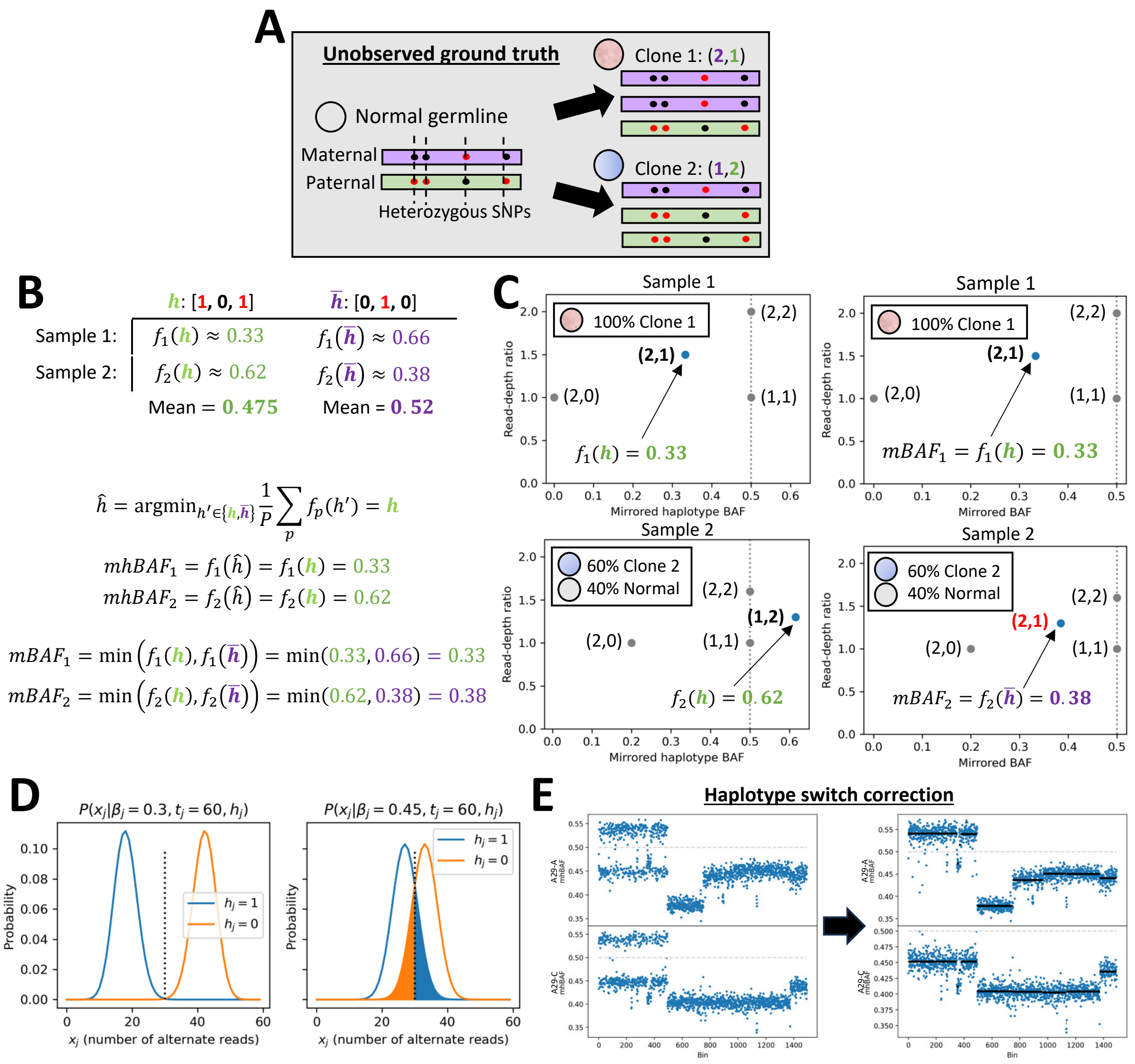

Fig. S8: Inference of minor haplotype BAF (mhBAF). A Single-bin example of ground truth haplotypes and clone haplotype-specific copy-number profiles that we would like to infer. **B-C** Numerical example of the difference between the mirrored BAF (mBAF) and HATCHet2's mhBAF. The mBAF chooses between haplotypes in a sample-specific manner, unlike the mhBAF which measures the same haplotype across all samples. Panel B shows the computation of each statistic; panel C visualizes the two statistics in the context of theoretical values of haplotype-specific copy-number states. **D** Distributions of alternate counts x_j for a single SNP j (in a single sample) with different phasing h_j and BAF β_j, under a simple binomial observation model. Shaded areas indicate regions where taking the $\arg\min$ would incorrectly assign the phase h_j for SNP j. **E** Example of haplotype switch correction performed on chromosome 7 in two WGS samples from prostate cancer patient A29 from Gundem et al. [12]. Black bars indicate segments of similar average mhBAF identified by HATCHet2's correction algorithm.

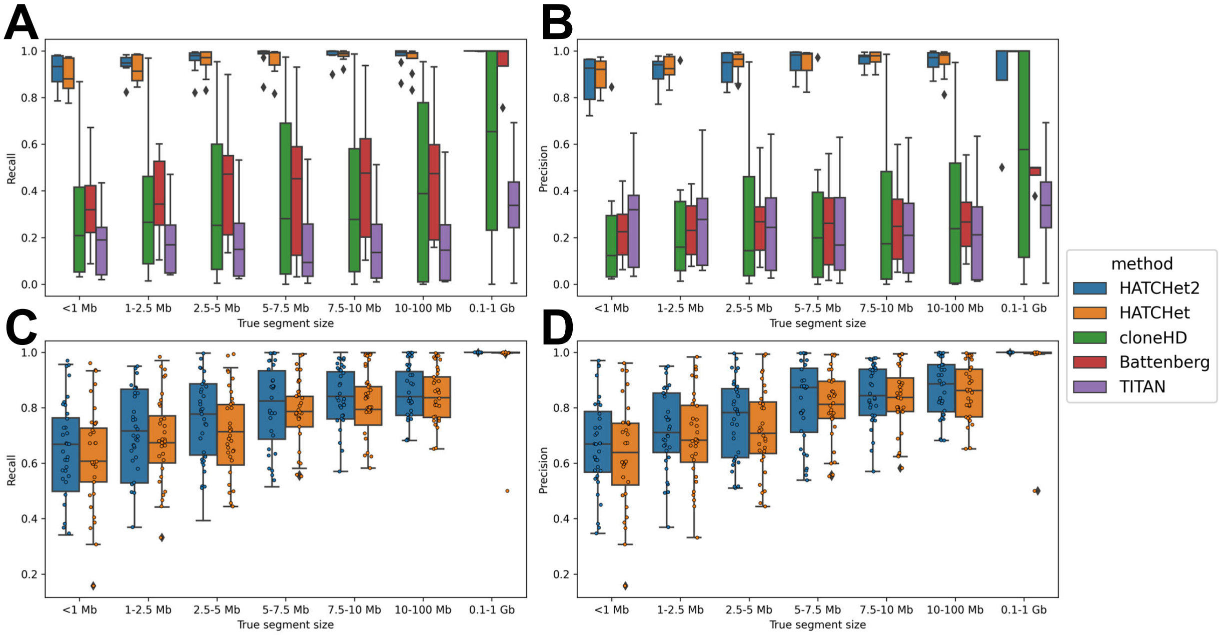

Fig. S9: Benchmarking HATCHet2, HATCHet [1], TITAN [4], cloneHD [3], and Battenberg [2] on simulated data by event size. **A-B** Results from all methods on 8 multi-sample datasets simulated using MASCoTE [1] measured by recall (A) and precision (B), stratified by the size of the ground truth simulated segment (x-axis). **C-D** Results on the same data with each sample run independently ($n = 32$ samples) measured by recall (C) and precision (D), stratified by the size of the ground truth simulated segment (x-axis).

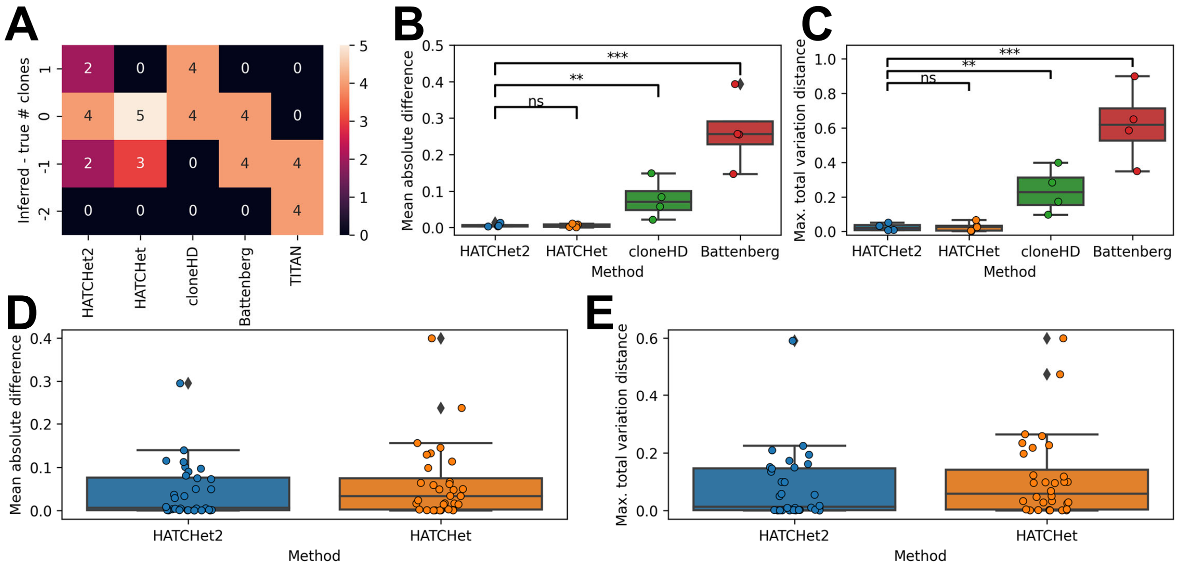

Fig. S10: Benchmarking HATCHet2, HATCHet [1], TITAN [4], cloneHD [3], and Battenberg [2] inference of clones and clone proportions. A-C Results from all methods on 8 multi-sample datasets simulated using MASCoTE [1]. In panels B-C, only those instances for which the corresponding method inferred the correct number of clones are evaluated. For panels B-C, asterisks indicate significance by unpaired t-test: $* = 0.01 \leq p < 0.05$, $** = 10^{-3} \leq p < 0.01$, $*** = p < 10^{-3}$. **A** Difference between the true and inferred number of clones from each method on the 8 datasets. **B** Mean absolute difference between the true and inferred clone proportions on multi-sample datasets. **C** Maximum total variation distance between the true and inferred clone proportions on multi-sample datasets. **D-E** Results from HATCHet2 and HATCHet [1] inferring clone proportions on the same data with each sample run independently ($n = 32$ samples), measured by clone inference error (D) and maximum total variation distance (E).

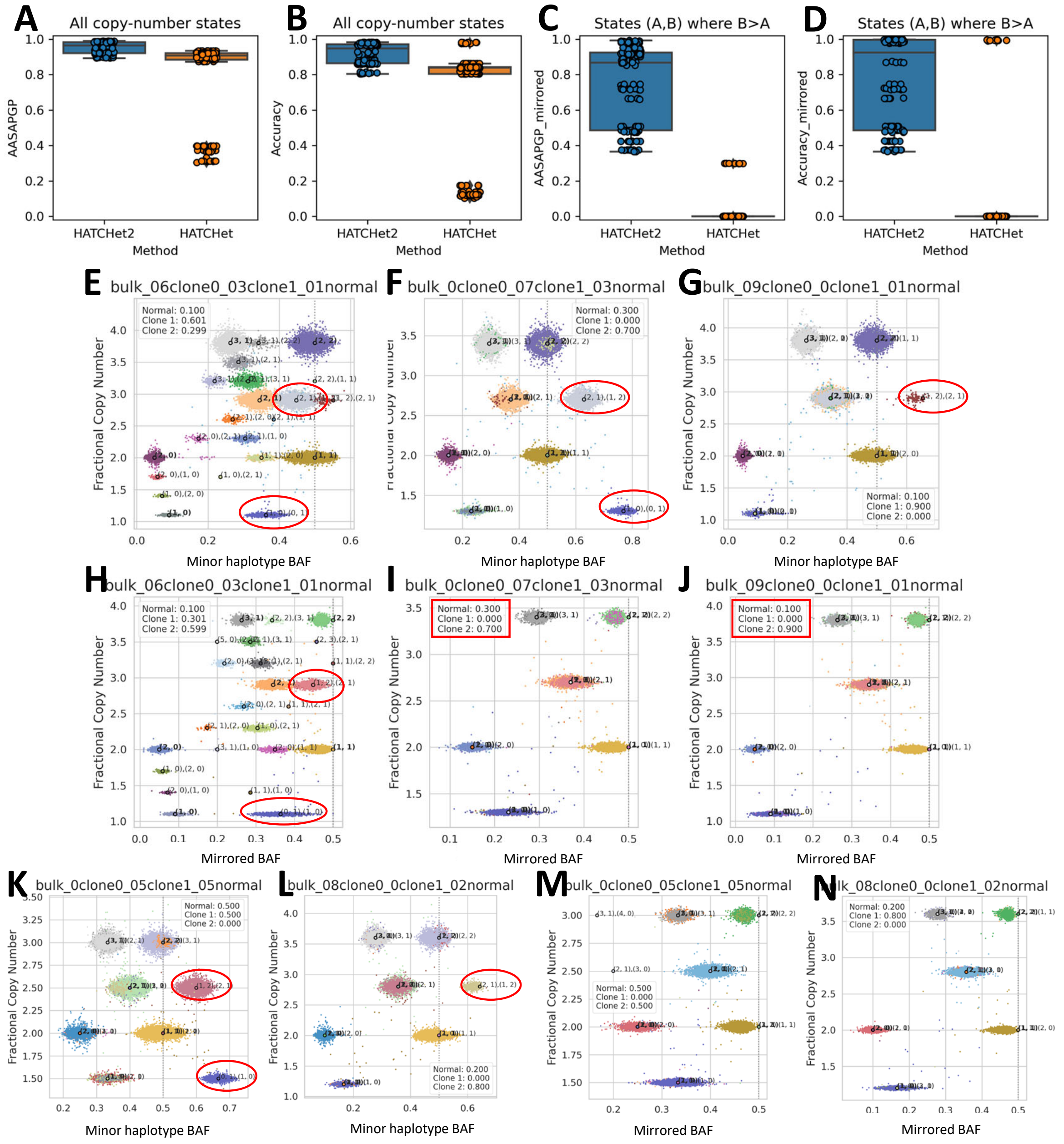

Fig. S11: Results of HATCHet2 and HATCHet [1] on simulated data with mirrored-subclonal CNAs. A-B Results from both methods on all copy-number states, measured by AASAPGP (A) and accuracy (B). **C-D** Results from both methods on mirrored-subclonal states only (i.e., only those states (A, B) where $B > A$), measured by AASAPGP (C) and accuracy (D). **E-J** Results from HATCHet2 and HATCHet on an example simulated dataset in which HATCHet achieves high accuracy on mirrored states (diploid genome 0, sample clone proportions 1, random seed 0). Panels E-G show the HATCHet2 results, and H-J show the HATCHet results. The title of each panel indicates the ground truth simulated clone proportions. Each point is a copy-number segment, colored by its assigned states. The labeled points with black outlines indicate the expected position of the assigned copy-number states given the inferred clone proportions. Red circles highlight some mirrored-subclonal copy-number states. **K-N** Results from HATCHet2 and HATCHet on an example simulated dataset in which HATCHet was not able to identify any mirrored states (diploid genome 0, sample clone proportions 2, random seed 0). Panels K-L show the HATCHet2 results, and M-N show the HATCHet results.

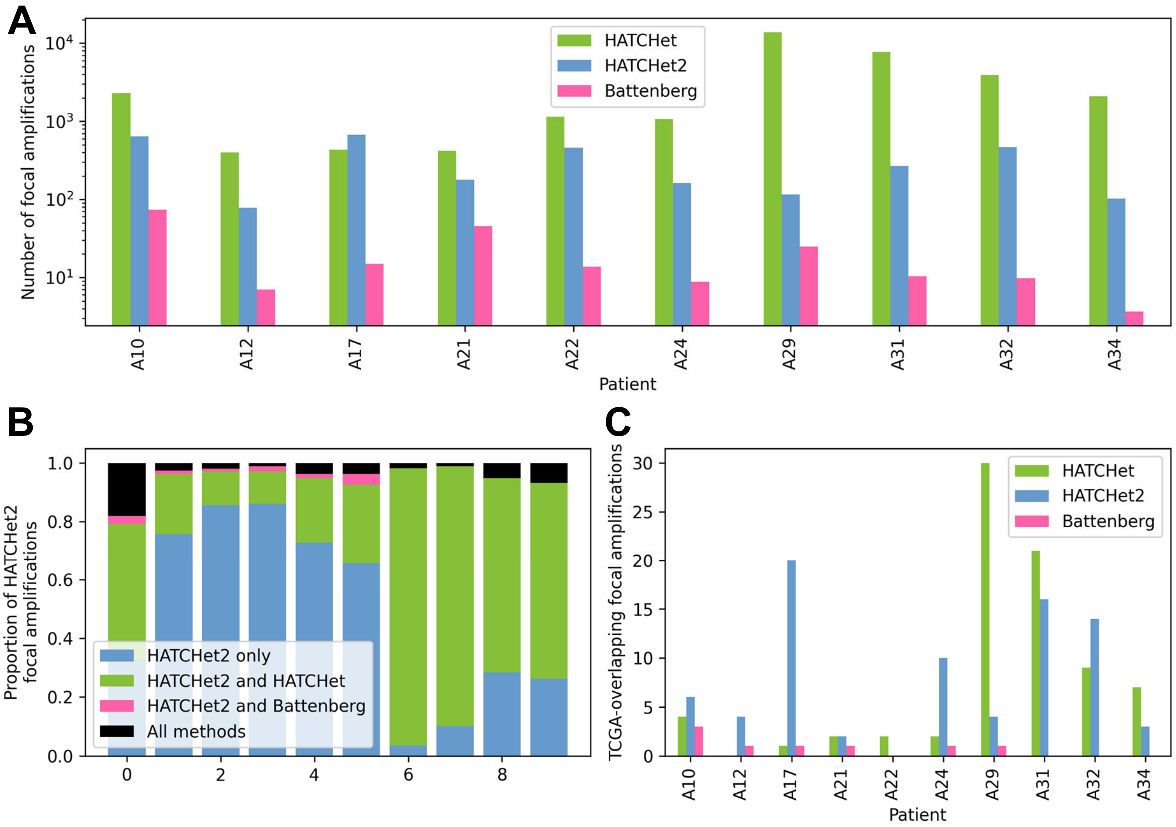

Fig. S12: Focal amplifications identified by HATCHet2, HATCHet [1], and Battenberg [2] on prostate cancer patients. **A** Number of focal amplifications – defined as segments shorter than 1 Mb with total copy number 4 or higher in at least 1 clone – reported by HATCHet2, HATCHet, and Battenburg on 10 prostate cancer patients from Gundem et al. [12]. **B** Breakdown of focal amplifications called by HATCHet2 in terms of overlap with those called by other methods. **C** Number of focal amplifications called by each method that overlap TCGA prostate cancer genes.

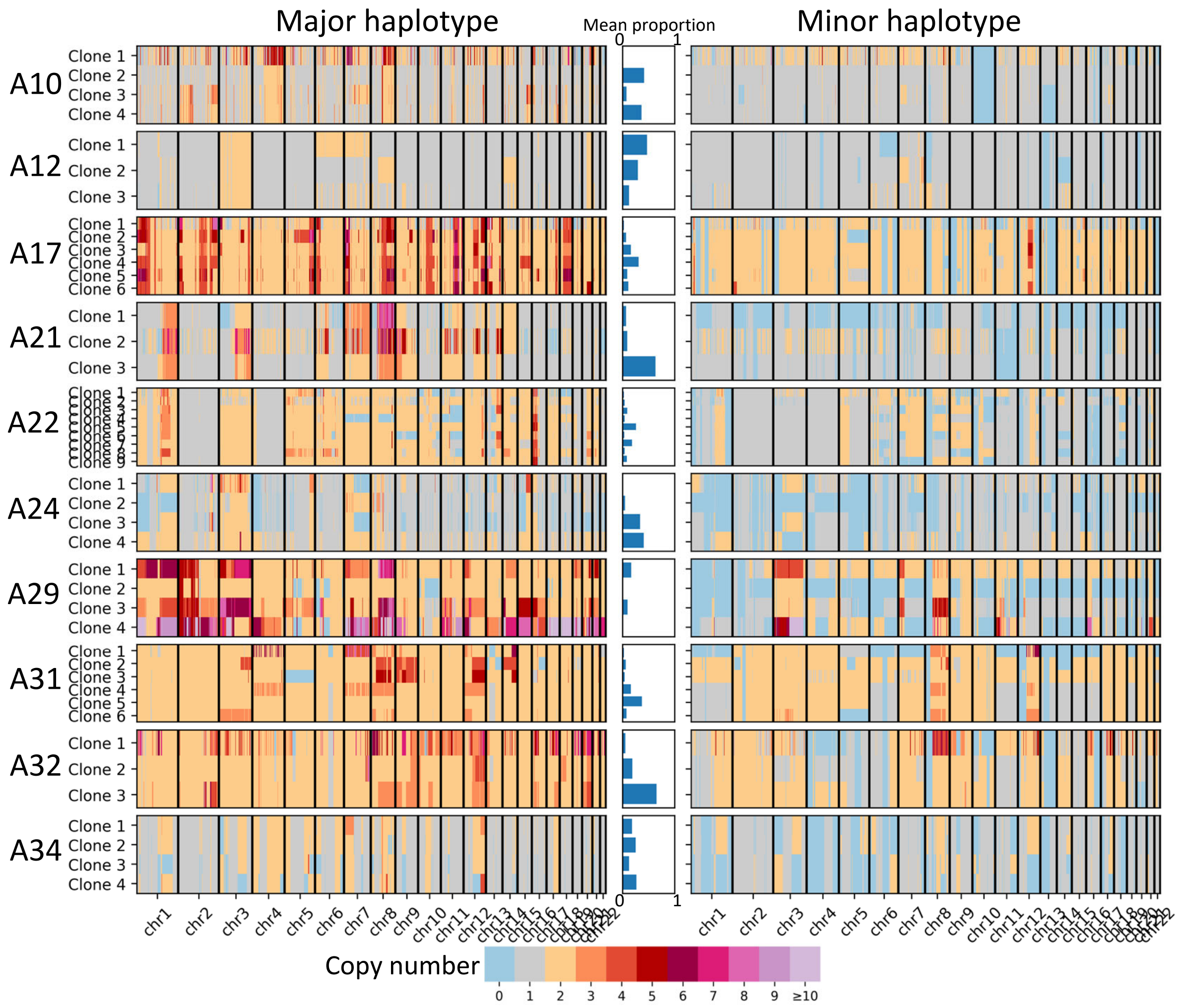

Fig. S13: Copy-number clones inferred by HATCHet2 on prostate cancer patients. Haplotype-specific copy-number profiles inferred by HATCHet2 on 10 prostate cancer patients from Gundem et al. [12]. The major haplotype for each clone is on the left, and the minor haplotype for each clone is on the right. The central column shows the average clone proportion for each clone across all samples (x-axis ranges from 0 to 1 for all patients).

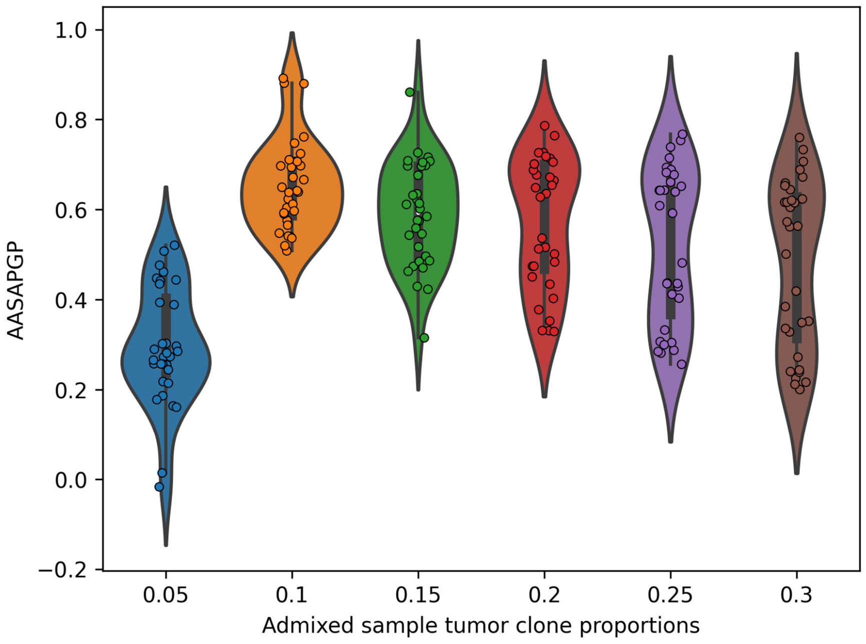

Fig. S14: Evaluation of HATCHet2 on simulated datasets with varying purity. Results on 191 simulated 2-sample datasets in which one "liquid" sample was of lower purity. Proportions of each clone ($n \in \{2, 3\}$ clones) in the liquid sample was equal (x-axis). Performance was evaluated using AASAPGP (y-axis).
